# Emission Estimation of On-Demand Meal Delivery Services Using a Macroscopic Simulation

**DOI:** 10.3390/ijerph191811667

**Published:** 2022-09-16

**Authors:** Maren Schnieder, Chris Hinde, Andrew West

**Affiliations:** The Wolfson School of Mechanical, Electrical and Manufacturing Engineering, Loughborough University, Loughborough LE11 3TU, UK

**Keywords:** PTV VISUM, macroscopic traffic simulation, on-demand meal delivery, collection and delivery points

## Abstract

While macroscopic simulations of passenger vehicle traffic within cities are now common practice, the integration of last mile delivery into a macroscopic simulation to evaluate the emissions has seldomly been achieved. In fact, studies focusing solely on last mile delivery generally focus on evaluating the delivery service itself. This ignores the effect the delivery service may have on the traffic flow in cities, and therefore, on the resulting emissions. This study fills this gap by presenting the results of two macroscopic traffic simulations of New York City (NYC) in PTV VISUM: (i) on-demand meal delivery services, where the emissions are evaluated for each OD-Pairs (i.e., each trip) and (ii) on-demand meal delivery services, where the emissions are evaluated for each link of the network (i.e., street). This study highlights the effect on-demand meal delivery has on the travelled distance (i.e., detours), congestion and emissions per km of every vehicle in the network, not just the delivery vehicles.

## 1. Introduction

Freight transport has often been ignored in urban policies [1]. One reason given is the lack of quantitative knowledge [1], especially a lack of information about the flow of goods [2]. While macroscopic traffic simulations of urban passenger transport are common practice, macroscopic simulations considering urban goods transport are still comparatively few [3]. Studies combining macroscopic traffic and emission simulations are rare for freight transport. On the other hand, the literature on combined macroscopic traffic and emission simulations for passenger transport is extensive [4]. However, urban goods transport is one of the causes of air pollution in residential areas [5,6,7] and the city centre [8,9,10]. The concerns about air pollution are becoming increasingly newsworthy, especially in urban environments due to the adverse effects on human health. The pollution levels in many cities around the world are considered dangerous [11]. The effect of pollution caused by traffic is more severe compared with the pollution caused by industry due to the close proximity of people to the source of transport pollution [12].

Schnieder et al. [13] have illustrated that the results of emission simulations are extremely sensitive to changing the simulation assumptions (e.g., road gradient, temperature, parking duration) or pollutants (e.g., CO_2_, PM_10_ exhaust, PM_10_ non-exhaust, and Well-to-Wheel emissions). The study presented in this paper adds to this narrative by highlighting the fact that delivery activities may also affect the emissions of other traffic within the network due to increased traffic congestion. In detail, the study contributes to the existing research and differs from previous publications as follows:

- Even though the relationship between emissions and congestion has been demonstrated in the literature [14], simulations of parcel delivery to lockers and meal delivery generally assume that the traffic flow will be the same regardless of the changes in the delivery service [13]. In contrast, this study considers that changes in the last mile delivery will affect the traffic flow, and therefore, the emissions. This study uses a similar methodology to calculate the emissions as in Tang et al. [12]. However, this study highlights the importance of adjusting emission factors depending on the traffic flow instead of only using average values. Other research has been focused on quantifying the total emissions, while this study highlights the increase in emissions per km. It is obvious that the emissions surge when the distance travelled increases. However, excessive congestion increases the emissions per km. Hence, the total emissions would be underestimated if a constant emission factor (i.e., g/km) is assumed.
- This study compares tour based with direct on-demand meal delivery services. In the first option, the meals are combined into a delivery tour while in the second option each meal order is distributed as a direct roundtrip between the restaurants and the customers.
- This study contributes to the research domain by analysing the effect the delivery network density (i.e., number of restaurants), as well as changes in the mode share for cars will have on the traffic flow and emissions.
- Emission estimation based on macroscopic traffic simulations can be calculated for each individual link (i.e., street, e.g., [4,14]), while emission simulations of delivery to parcel lockers are usually calculated per trip (see literature review in [13]). The study links both methods by comparing link-based emission estimates with OD-pair (i.e., origin and destination) based emission estimates.
- This study also highlights the difference in the results if the average speed is used as an explanatory variable for emission factors (e.g., in [12], referred to as OD-pair based) instead of using traffic flow and road type (referred to as link based). This is an important consideration given that 50 km/h might be the free-flow speed in a city but on a motorway, 50 km/h would indicate a saturated traffic flow.
- Other authors, such as Heldt et al. [15], simulate passenger transport in one model and deliveries in another model (i.e., no interaction between both). In contrast, this study optimises the delivery trips independently to ease the simulation but then aggregates the delivery trip demand in addition to the passenger vehicle demand into the same macroscopic simulation.

The study focuses on on-demand meal delivery, where cooked meals are ordered from a restaurant and delivered to the customer. This should not be mistaken for meal delivery (e.g., HelloFresh, https://www.hellofresh.co.uk (accessed on 11 September 2022)) where the customer receives the ingredients for a few meals in a parcel to cook themselves at a later time, or delivery of cold foods (i.e., sandwiches). While the market for on-demand meal deliveries is growing, the expectation of customers to receive the meal exactly when they want to eat it and shortly after it has been cooked presents a challenge for delivery companies [16]. Due to this, on-demand meal delivery is one of the more challenging delivery goods. Parcels for example can be delivered to lockers when the customer is not at home, which cannot be conducted with cooked meals that are delivered warm. To achieve the required flexibility of the delivery service, on-demand meal delivery platforms commonly rely on crowd sourced delivery couriers who usually ride a bicycle or drive a car. Recently, the interest in using sidewalk autonomous delivery robots (SADRs) for on-demand meal delivery is increasing [16]. Two types of delivery services have emerged, platform-to-consumer (e.g., Deliveroo and UberEATS) and restaurant-to-customer (e.g., Domino’s). The latter is the focus of this paper.

The remainder of the paper is structured as follows. Initially, the results of a systematic literature review are presented. Then the methodologies and results of the first study are illustrated (i.e., on-demand meal delivery services, where the emissions are evaluated for each OD-Pair (i.e., each trip)). Next, the methodologies and results for the second study are detailed (i.e., on-demand meal delivery services, where the emissions are evaluated for each link of the network (i.e., street)). Finally, the results are discussed and conclusions are drawn.

## 2. Literature Review

While PTV VISUM and PTV VISSIM are the most widely used traffic simulation tools [17], the studies using PTV VISUM for freight simulation are comparatively few as shown in the following literature review, which has been conducted using the PRISMA guidelines [18]. The search was conducted in May 2021 and in July 2022. The search in July 2022 has been conducted to identify any papers which have been published since the search in 2021. The keywords *(“Visum”) and (“HGV” OR “logistics” OR “freight” OR “last mile delivery” OR “On-demand meal delivery”)* have been used to find relevant papers in ScienceDirect. In contrast with the search on ScienceDirect, the search on Scopus was limited to abstract, title and keywords. Hence, a longer keyword was chosen given that not all papers mention VISUM in the abstract: *(“Macroscopic traffic simulation” OR “VISUM”) AND (“goods” OR “HGV” OR “logistics” OR “freight” OR “last mile delivery” OR “On-demand meal delivery”)*. The search was limited to papers published since 2015. The search identified eight papers published on Scopus (i.e., seven published before 2021 and one in 2022). A total of 80 papers have been identified on ScienceDirect (i.e., 66 in the search in May 2021 and 14 papers have been published since then). After removing the duplicates, the full text of the papers been screened to identify papers that meet the following inclusion criteria:

- Conducted a traffic simulation in PTV VISUM.
- Focus amongst other things on freight transport on roads in their simulation and not on freight transport on rail/cargo trains or intermodal transport.

The number of papers not relevant to the focus of this research is large given that papers which conduct a simulation in PTV VISSIM mention PTV VISUM or they conducted a simulation in VISUM that does not focus on freight transport. All relevant papers (*n* = 13) are summarised in Table 1.

Perera et al. [19] created a simulation of Melbourne and surrounding areas to evaluate the effect of toll charging schemes on emission costs. The goal of the study was to minimise the emissions while generating revenue for investors through road tolls. They used OpenStreetMap street network data and estimated the OD-matrix based on Average Annual Daily Traffic (AADT). The authors were able to create an OD-matrix which, once assigned to the network, resulted in a 92% correlation for all links.

Perera et al. [20] used the same methods as in [19] to create a simulation of Melbourne in VISUM to estimate the total costs of freight transport (i.e., economic, social and environmental) as well as to identify options to minimise the total cost.

Gorin et al. [21] described a four-step method to create a freight simulation in PTV VISUM but did not present any results. They estimated the trip generation and trip attraction based on the number of workplaces for each vehicle capacity.

Gupta et al. [2] used a four-step model to create a simulation of freight transport in a medium sized city in India. The focus was on evaluating the status quo of freight transport as opposed to simulating scenarios.

Tang et al. [12] simulated four real scenarios and one hypothetical scenario in PTV VISUM to compare different HGV management strategies. They considered various pollutants including PM (non-exhaust) emissions but disregarded cold-start emissions. A variation of the emission simulation methodology used in Tang et al. [12] has been applied in this study. They concluded that while a bypass improved the traffic flow in the city (i.e., 400–500 cars/h and 100–200 HGVs/h less), the increased travel distance (i.e., cars: from 917,713 km to 917,663 km; HGV: from 72,755 km to 84,605 km) also raised the total emissions (i.e., from 719.05 kg to 775.36 kg).

While the previous studies used PTV VISUM to simulate freight transport within a city, the following studies simulate freight delivery across larger regions or entire countries.

Martino et al. [22] and Fiorello et al. [23] used PTV VISUM as part of an integrated modelling system that includes three main blocks (i.e., transport, economy and energy model). The transport model includes a passenger demand model, a freight demand model and a network model/assignment model. The simulation covers EU28 and candidate and potential candidate countries. They differentiate between two matrixes for their freight model: (i) a production-consumption (PC)-matrix of trade (i.e., trade forecast), which is influenced by the economy model and (ii) OD-matrix of shipments which represents the actual transport activity.

Grebe et al. [24] described the process they would apply to create a transport model for Austria. The goal is to estimate one demand matrix for passenger vehicles and one for freight transport and assign both to a network simultaneously in a quasi-dynamic procedure.

Roider et al. [25] focused on improving freight demand data estimation by combining multiple data sources. This study used various data sources including surveys, statistics and road toll data in Austria. They applied the VStromFuzzy matrix estimation to correct the OD-matrix using traffic count data.

Savadogo et al. [26] compared the environmental impact of daytime delivery with out-of-hours delivery. The total traffic has been derived by aggregating a passenger (SIMBAD) and a freight (FRETURB) matrix. They used a private car equivalency factor (PCE) to convert freight movements into private motor vehicles (PMV). The freight vehicles are light goods vehicles (LGVs) and rigid or articulated heavy goods vehicles (HGVs). They concluded that depending on the share of out-of-hours deliveries and pollutants, the emissions can be reduced by up to 7.6% and an estimated € 4.25 million in annual environmental and social costs can be saved.

Jacyna et al. [27] created a simulation of Poland which considered freight transport and passenger transport using trains, buses, trucks and lorries and analysed the emissions of the status-quo. They concluded that their model can be used in the future to assess the impact infrastructure development decisions and policies have on emissions.

Gnap et al. [28] used VISUM to estimate the accessibility of an intermodal logistics terminal by road in its current and future state. They used the transport model of the Žilina self-governing region for their simulation. They concluded that accessibility is acceptable as long as there are no major disruptions due to traffic accidents and other incidents.

Binh [29] created a simulation in VISUM to visualise freight movements in northwest Vietnam. They simulated the cargo flow between 14 zones using forecasted data. They used the simulation to propose an optimal location for four logistics centres (i.e., Hanoi, Phu Tho, Yen Bai and Lao Cai).

As can be seen in Table 1 most studies use either survey data, socio-economic data, traffic count data or OD-demand matrixes from a different model to estimate the traffic demand.

Some studies do not specify whether they use link-based calculations or OD-based calculations, e.g., Savadogo et al. [26] stated that they used the distance and time from the VISUM simulation as input to the emission calculation (which might imply that they used an OD-Pair based calculation). Other studies state clearly that they calculated the emissions for each link (e.g., [27]) or for the share of the total distance ([12]).

## 3. Methods: On-Demand Meal Delivery (OD-Pair Based)

### 3.1. Overview

**Figure 1:**
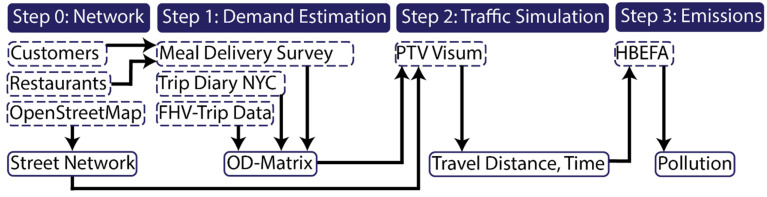
Study methodology (on-demand meal delivery (OD-pair)). For hire vehicles (FHV).

All simulations in this study have been implemented in PTV VISUM 20.01 Expert (Academic licence) a macroscopic traffic simulation software that is one of the leading commercial software systems for transportation planning [1,19]. A modified version of the four-step methodology proposed by Savadogo et al. [26] has been adopted in this study. Figure 1 shows the general study methodology which has been adjusted for each individual study.

All of the figures have been produced using the Python libraries seaborn [30] and matplotlib [31] as well as PTV VISUM 20.01 (Createor: PTV Planung Transport Verkehr GmbH, Karlsruhe, Germany).

### 3.2. Traffic Network and Transport Demand by Cars

Step 0: The OpenStreetMap (OSM) street network of New York City (NYC) [32] has been used, which consists of 569,098 links and 121,261 nodes. OSM street network data are commonly used in PTV VISUM (e.g., [19,24]) as a street network.

Step 1: The demand data for cars in this simulation have been estimated based on the yellow and green taxi as well as “for hire vehicle” (FHV) trips in NYC from 2017 to 2019 [33] and based on the Mobility Survey of NYC [34]. Only trips that are between 1 min and 4 h long and start between 6 am and 9 pm have been included given that the focus is on simulating daytime traffic movements. Using FHV data is not a viable option in most cities. However, FHV trips account for between a third and more than half of the vehicle traffic on selected streets within Manhattan and surrounding areas [35]. The traffic volume of buses, trucks and light goods vehicles (LGV) account for a quarter or less on these streets. However, the taxi and “for hire vehicle” volume in all other Boroughs can reasonably be expected to be less. To avoid underrepresenting trips to/from/within these other Boroughs, each OD-pair of the FHV trip OD-matrix has been assigned a weight according to the relative volume of trips by private cars or for hire vehicles between Boroughs based on the Mobility Survey of NYC [34]. The equation used to weight the demand for each OD-pair is shown in Equation (1):

(1) $$D_{o,d}={\;\mathrm{FHV}}_{o,d}*\frac{\sum_{o=0}^{O}\sum_{d=0}^{D}{\mathrm{FHV}}_{o,d}}{{\mathrm{FHV}}_{o,d}}*\frac{S_{s,e}}{\sum_{s=0}^{S}\sum_{e=0}^{E}S_{s,e}}$$

where, $D_{o,d}$—Weighted demand from FHV zone o to d

${\mathrm{FHV}}_{o,d}$—FHV demand from FHV zone o to d

$S_{s,e}$—Demand from survey zone s to e

O,D—Number of FHV zones

S,E—Number of survey zones

The FHV zones and survey zones have been mapped to each other using QGIS [36]. It would have been possible to use only the trip data between the 10 zones from the Mobility Survey of NYC [34] as the OD-matrix. However, ten zones are not enough to achieve a meaningful macroscopic simulation of on-demand meal deliveries in NYC. Using the FHV trip data as a basis for an OD-demand matrix and then adding weights to each OD-pairs based on survey data allowed for a much more precise OD-matrix estimation. Each of the 263 zones in the FHV trip data set and the corresponding demand have been split into three zones, otherwise, the relatively large size of the zones and the low number would have had a negative effect on the precision of the simulation.

The authors acknowledge that the chosen approach is not the standard way to create an accurate virtual representation of the traffic flow in NYC. For example, real traffic counts can be used to calibrate the demand (i.e., OD-matrices). This step is necessary given that traffic simulations are usually based on a small amount of data, which cannot adequately represent complex and local patterns of transport flows [24]. However, this approach was the most suitable one for the study aim given that this simulation uses the origin and destination data of Taxi and FHV as an input which account for 50% or more of the traffic volume on selected streets that are within Manhattan [35]. Note, this share is lower in the rest of NYC. Furthermore, the aim of this study is to compare multiple traffic demand levels. Hence, for the aim of this study, it is not an issue whether the macroscopic simulation is an accurate virtual representation of NYC or which demand level represents the true demand level. The study highlights the importance of implementing transport policies early before the negative effects are visible. Hence, most of the scenarios in this simulation may not occur in real life, and therefore, no real-life traffic count data are available to calibrate these scenarios. The focus is more on creating results that emphasise the importance of adjusting the emission factor based on the traffic congestion, which can be generically applied to other cities, as opposed to accurately representing one real-world city. To emphasise this, the study does not focus on measuring the exact total emissions but rather analyses the effects, relationships and changes.

### 3.3. Delivery Network and On-Demand Meal Delivery Demand

Step 0: The list of all addresses in NYC [37] has been used as the location of customers and the zones which contain a restaurant have been randomly selected.

Step 1: In other studies, OD-matrices for urban freight transport are commonly estimated based on geo-economic data (i.e., employment, shops) or traffic count data [1]. In this study, the demand for meal delivery has been estimated based on a dataset of all addresses [37], the population density in NYC [38] and the probability that a resident in a specific neighbourhood receives a meal delivery on any given day based upon a survey [34] of residents in NYC. A list of orders has been created based on a binomial random number generator [39] in the Python programming language (*n* = number of residents at an address, *p* = probability that a resident orders a meal). The same methodology to estimate the demand for on-demand meal delivery has been used in [16]. Each customer receives the order always from the nearest restaurant, which is calculated using the nearest neighbour algorithm in QGIS [36]. No private car equivalency factor (PCE) was required given that all meals are delivered using a private motor vehicle (PMV).

### 3.4. Scenarios

Three scenarios have been simulated: (1) varying traffic demand, (2) varying mode share and the number of restaurants and (3) tour-based delivery service.

Scenario 1: The baseline traffic volume has been increased and decreased by 20% and 40%, respectively. No meals have been delivered or picked up by the customer in this scenario

Scenario 2: Each meal order is delivered as a direct round trip from the restaurant to the customer’s zone. It has been assumed that either 0%, 20%, 40%, 60%, 80% or all meals are delivered by PMV. The number of restaurants varied between 1, 2, 4, 8, 16, 32, 64, and 128. The passenger transport volume is always the same as the baseline of scenario 1. The meal delivery demand and the passenger transport demand (i.e., the baseline traffic described earlier) are aggregated and assigned to the network.

Scenario 3: This scenario was only simulated for the case when 128 restaurants exist. All meals are delivered by a tour-based meal delivery service. The delivery tours have been optimised using a locally hosted Open Source Routing Machine (OSRM) [40] using OpenStreetMap (OSM) street network data [32]. Every 15 min, 30 min and 60 min a delivery van starts a delivery tour from each of the 128 restaurants. It is assumed that this tour-based delivery service operates 24 h per day and the demand for on-demand meal deliveries is homogeneously split across delivery time slots throughout the day. Each delivery van travels on a delivery tour to all customers allocated to that restaurant. For example, a delivery van from a restaurant in zone A could deliver in a specific delivery slot to three customers in zone A, four customers in zone B and five customers in zone C. The optimal order of drop-off points are determined by the OSRM (e.g., zone A first, zone B second and zone C last). The delivery van would first complete three internal trips within zone A to deliver the meals. Then the delivery van drives to zone B to deliver a meal before completing three internal delivery trips within this zone to deliver the three remaining meals. Finally, the delivery van drives to zone C where the same process is repeated before driving back to zone A (i.e., restaurant). The resultant demand matrix, of this simplistic example, is shown in Table 2.

**Figure 2:**
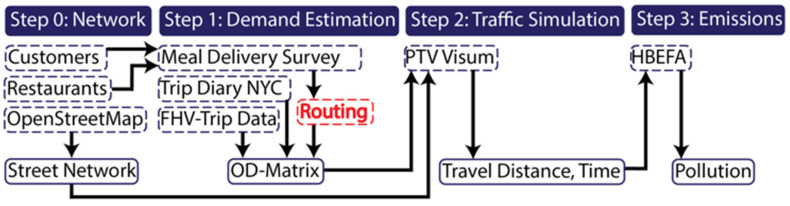
Study methodology (On-demand meal delivery–tour based (OD-pair)).

In short, an OD-matrix has been created based on the delivery tours. Trips within each zone to deliver to multiple customers within that zone have been considered. As before, the traffic from the delivery is aggregated together with the baseline demand. Figure 2 illustrates the methodology. 

### 3.5. Assignment

Step 2: Instead of creating the scenarios by hand in VISUM, the COM-API has been used to run Python scripts to automate the scenario calculation (i.e., loading the correct OD-Matrixes into VISUM, start the procedure sequence and extracting the results). Using Python scripts is a consistent and transparent way to implement and automate functionalities into VISUM that are not part of the standard software [23]. For each case, the meal delivery OD-matrix and the passenger vehicle OD-matrix have been aggregated and assigned to the network using the equilibrium assignment.

### 3.6. Emission Modelling HBEFA

Step 3: The emission simulations methodology has been performed similarly to Tang et al. [12]. The only difference is that Tang et al. [12] used COPERT 4 as the emission database while this study uses the Handbook Emission Factors for Road Transport (HBEFA 4.1) [41]. HBEFA provides emission factors for a variety of traffic situations, temperatures, street types and drive train types. The emission factors considered in this study are total hydrocarbons (HC), carbon monoxide (CO), nitrogen oxides in NO_2_ equivalents (NO_x_), particulate matter (PM_10_), PM_10_ caused by non-exhaust emissions, such as tyre wear (PM_10_ (non-exhaust)) and total carbon dioxide including biofuel share CO_2_ (total) [41]. It should be noted that the HBEFA only includes vehicle activity data for five European countries and has not been validated for use in American cities. Hence, the emission factors may not accurately represent the emissions in other countries [42]. Nevertheless, HBEFA has been used given that it is one of the most frequently used emission models [43] and using European data in other continents are frequently published by other authors (e.g., emission costs in [19]).

The vehicle used to deliver the meals is assumed to be a PMV. Given that NYC is generally flat, with the highest point in NYC being only 124 m above sea level, a gradient of 0% has been chosen. A four-stroke petrol engine with Euro-5 has been chosen as an emission concept as no detailed fleet composition data in NYC were available.

For simplicity, all vehicles in NYC are assumed to be used 24/7. Hence, no cold start emissions are included in this simulation. Other authors also disregarded the cold start emissions in their simulation (e.g., [12]). The traffic situation has been chosen according to the average speed for every OD-pair. Given that most streets in NYC have a speed limit of 50 km/h (based on the OpenStreetMap network: 6% < 49 km/h, 64% 59 km/h, 30% > 51 km/h, roads without car access are excluded) and “URB/Trunk-City/50/? has been chosen as the street type.

### 3.7. Limitations

Scenarios are simulated regardless of whether they are observed in reality (see Savadogo et al. [26]). The passenger transport demand stays constant across scenarios 2 and 3, and therefore, induced demand or model shift is not considered reality (see Savadogo et al. [26]).

## 4. Results: On-Demand Meal Delivery (OD-Pairs)

### 4.1. FHV Trip Data

**Figure 3:**
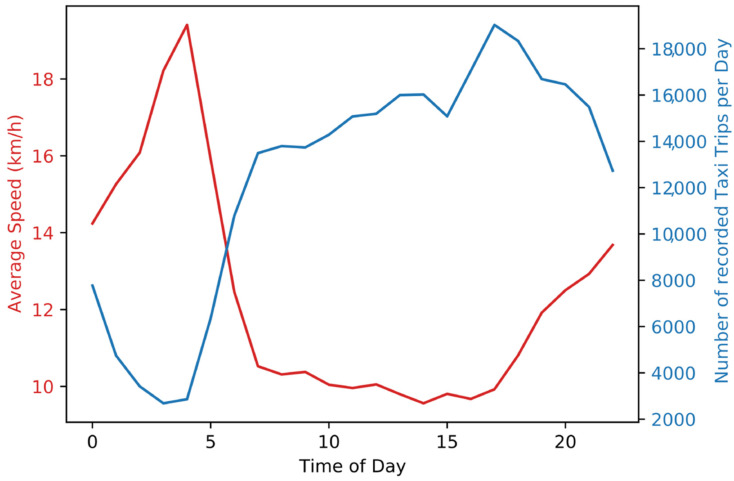
Average speed and number of recorded yellow and green taxi trips for different times of the day (data set: [33]).

Figure 3 shows the average speed of the FHV trips and the percent of daily FHV trip volume based on the Taxi and FHV trips in NYC between 2017 and 2019 [33]. The average speed is for most parts of the day around 10 km/h. Only during the night and evening does the average speed increase and peak at 19 km/h. The mean speed is 11 km/h. The FHV trip volume is high throughout the day and early at night.

### 4.2. Scenario 1

**Figure 4:**
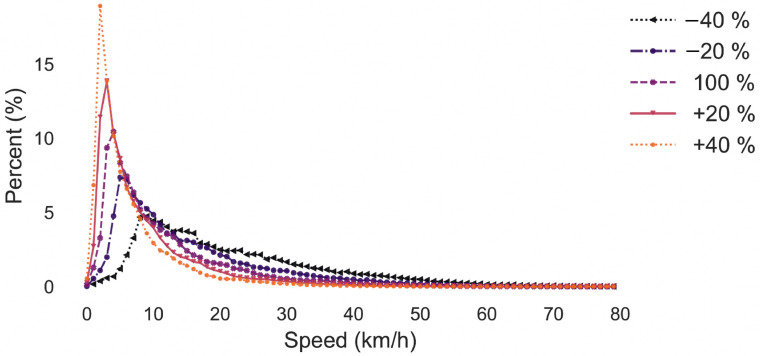
Speed for various demand levels (scenario 1). Percent refers to the share of trips which have a specific average speed.

The average speed per trip for the five cases (i.e., baseline traffic volume and 20% and 40% increase/decrease) in scenario 1 is shown in Figure 4. The mean average speed ranges from 7 km/h (+40% demand) to 22 km/h (demand −40%). The range of average speeds seen in the macroscopic simulation is comparable to Figure 3. Obviously, this does not necessarily mean that the simulation is a realistic representation of NYC. It only means that the results are within the range of what can be expected from a macroscopic simulation of a large city and is, therefore, sufficient for the aim of this study.

**Figure 5:**
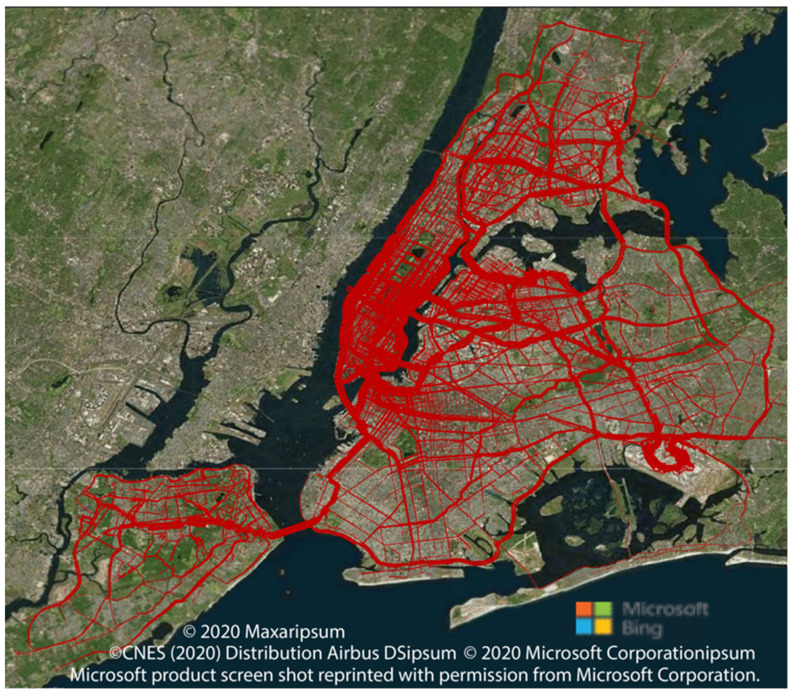
Traffic flow (baseline scenario 1) (created with PTV VISUM 20.01, printed with permission from PTV Planung Transport Verkehr GmbH).

Figure 5 shows the traffic flow for scenario 1 (baseline). As expected, the traffic flow is the highest in Manhattan CBD and the Midtown Core. Note: the traffic flow towards John F. Kennedy International Airport is higher than expected for typical journeys. This is one of the problems with using Taxi data as an input.

**Figure 6:**
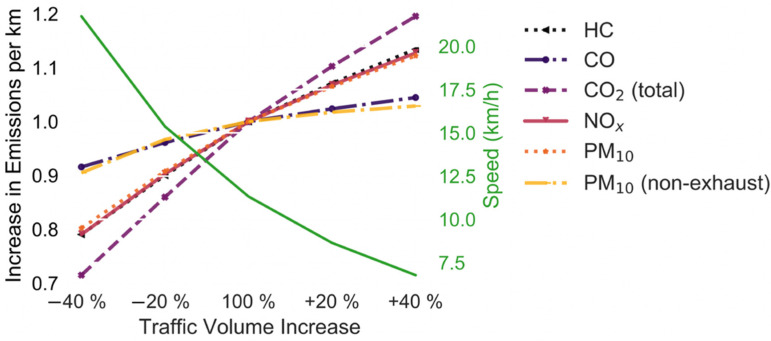
Increase in emissions per km (scenario 1) (HC, NO_x_, and PM10 are overlapping).

Figure 6 shows the increase in emissions per km and the change in speed due to the increased traffic volume. Most emission factors increase or decrease by 10% to 30% when the traffic volume changes by 40%. Only the PM_10_ (non-exhaust) emissions and CO emissions are not affected much by an increase in the traffic volume. This figure indicates that using the same emission factor regardless of the traffic volume or average speed would yield incorrect results. The average speed in NYC varies throughout the day from 9.5 km/h to 19.4 km/h. Hence, simulations of NYC should consider that the emission factors increase and decrease by approximately 5–10% depending on the pollutants chosen throughout the day. This again highlights the advantages of delivering parcels at night to lockers as shown in [13].

### 4.3. Scenario 2

**Figure 7:**
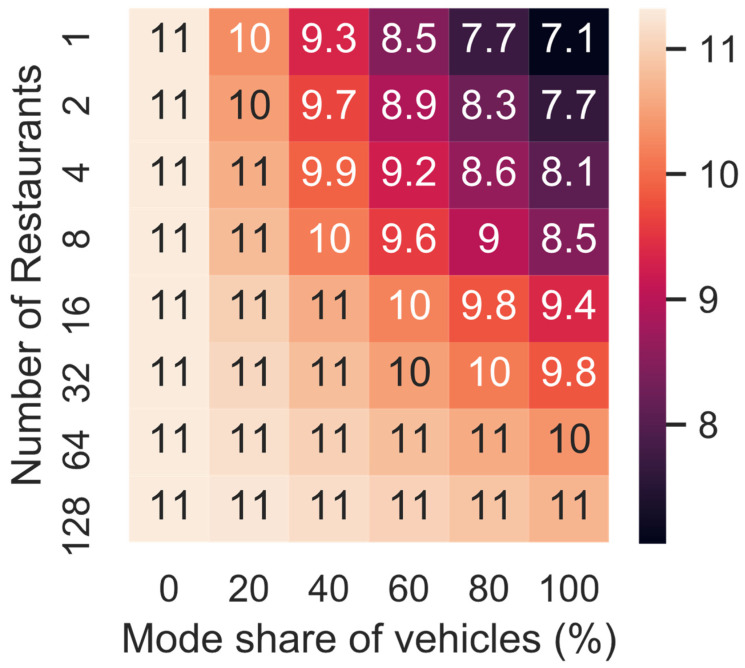
Average speed for varying mode share and number of restaurants.

Figure 7 illustrates how the average speed changes depending on the mode share (i.e., share of the meals delivered by a PMV) and the restaurant network density (i.e., number of restaurants in the network). Even if all meals are delivered by a PMV, the average speed does not increase given the distance between the restaurant and the customers is sufficiently short (i.e., 128 restaurants, the bottom row of the heatmap in Figure 7). This could be caused by the fact that short trips are more likely to go through residential neighbourhoods, which are not as congested as the main roads. Reducing the number of restaurants increases the traffic volume, and therefore, reduces the average speed as the trips are longer, and therefore, more likely to utilise main roads. If 60% of all meals were delivered by PMV this would reduce the average speed by 2.5 km/h if all meals are delivered from one restaurant.

**Figure 8:**
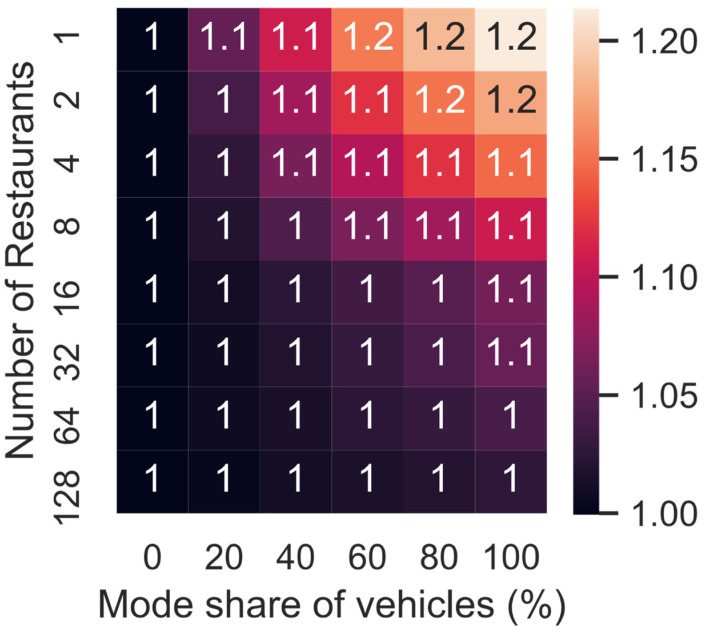
Increase in the CO_2_ emissions per km for varying mode share and number of restaurants.

Figure 8 shows the increase in the CO_2_ emissions per km due to the change in the number of restaurants and mode share. If meals are delivered from the same restaurant by a PMV, the CO_2_ emissions per km are 21% larger than in the baseline (i.e., no meal delivery). This increase is 14% for PM_10_, 3% for PM_10_ (non-exhaust), 14% for NO_x_, 15% for HC and 7% for CO.

If all meals were delivered by PMVs, the total distance travelled by all vehicles across the network ranges from 21,264,622 km (128 restaurants) to 29,284,313 km (1 restaurant). Hence, the additional emissions, caused by reducing the number of restaurants, are substantial. The increase in distance is mainly caused by an increase in the distance travelled between restaurants. However, the distance travelled between zones also increases by 8.3% (1 vs. 128 restaurants) on average possibly due to detours to avoid traffic (i.e., implemented using the traffic assignment procedure in PTV VISUM). In summary, increasing the share of meals delivered by PMVs or increasing the distance between restaurants increases the total distance travelled by 37% (including the increase in the distance which is possibly due to detours to avoid traffic), increases the emissions per km by 3% to 21% depending on pollutant and reduces the average speed throughout NYC by up to 4 km/h.

### 4.4. Scenario 3

While the meals in the previous scenario are delivered as a direct round trip to the customer, in this scenario the customer receives the delivery whenever the delivery tour arrives at the zone. Each delivery vehicle visits between 3 and 25 zones (mean: eight zones). On average it takes 20 min to deliver the meals (excluding the travel time within each zone to visit multiple customers or to hand over the meals). The average speed and the emissions change by less than 8% and 0.3%, respectively, when the delivery tour frequency (i.e., every 1 h vs. 15 min) is changed. The emissions produced in addition to the emissions in the baseline scenario are eight to nine times higher for direct delivery from one restaurant compared with a tour every 15 min from 128 restaurants. The emissions are three to four times higher for direct deliveries from 128 restaurants compared with a delivery tour every 15 min from 128 restaurants.

## 5. Methods: On-Demand Meal Delivery (Links)

### 5.1. Traffic Network, Demand and Assignment

**Figure 9:**
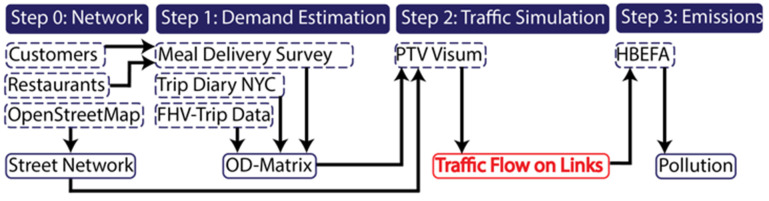
Study methodology (On-demand meal delivery (Links)).

The same traffic network, traffic demand and traffic assignments as in the on-demand meal delivery (OD-pairs) simulation have been used in this scenario. The only difference is that the traffic flow on links has been used to calculate the emissions instead of calculating the emissions based on OD-pairs (i.e., trips) (Figure 9).

### 5.2. Emission Modelling HBEFA

The emission calculation methodology in this simulation is calculated based on links while Tang et al. [12] calculated the emissions based on the share of the total distance. The emission factors in Tang et al. [12] only vary based on the vehicle class and average speed. However, researchers advise against using only the average speed as an explanatory variable for vehicle emissions, especially in congested cities [44]. While an average speed of 50 km/h would represent free-flow conditions in a city, the same average speed would be considered saturated (i.e., congested) on an urban motorway with a maximum speed of 100 km/h [45]. The emission factors for “URB/MW-City/100/Satur.” are between 4% and 6 times higher than “URB/Trunk-City/50/Freeflow” depending on the pollutant. To highlight this problem, this study compares: (i) the emission factors based on the average speed of an OD-pair (i.e., trip) with (ii) the emission factors according to the traffic flow and street type. Tang et al. [12] only considered three average speed categories, whereas this simulation considers five traffic flow categories.

The settings for the emission calculation are the same as in the previous example (i.e., on-demand meal delivery (OD-pairs)). The only difference is that both the traffic situation and the street type have been chosen according to the speed limit of the street and the average speed on that street. Note, in the previous simulation it was not possible to select the emission factors according to the street type given that the vehicles travel on multiple street types during each trip (i.e., OD-pair).

## 6. Results: On-Demand Meal Delivery (Link Based)

### 6.1. Scenario 1

#### 6.1.1. Link-Based and One Street Type vs. OD-Pair Based

As a comparison, the same street type (i.e., “URB/Trunk-City/50/”) is used for both emissions calculations (i.e., link-based and OD-Pair based). In this case, the link-based emissions are 8–18% smaller than the OD-pair based emissions Table 3. This is the result of the variation in the calculated average speed as explained before.

#### 6.1.2. Link-Based and Correct Street Types vs. OD-Pair Based

In Table 4, the street type has been selected as explained in Section 5.2. The emissions are higher for CO (i.e., up to 48%) and lower for all other emissions (i.e., up to 27%) when the correct street type has been selected for each link compared to when the street type is assumed to always be “URB/Trunk-City/50/”. One reason is that 64% of all links have a speed limit of 50 km/h (i.e., “URB/Trunk-City/50/”). Hence, the traffic on a “URB/MW-City/100/” street would be considered “Heavy” if the average speed is between 66 km/h and 83 km/h, while the same average speed would be considered as free-flow on an “URB/Trunk-City/50/” street. Hence, the emissions are higher. This effect is influenced by the fact that free flow traffic on a street with a maximum speed of 40 km/h or 30 km/h would be considered heavy or saturated traffic, respectively, if all streets were assumed to be “URB/Trunk-City/50/”. However, only 6% of all links have a speed limit of less than 50 km/h, while 30% have a speed limit of more than 50 km/h.

This comparison shows that calculating the emissions produced on each link vs. calculating the emissions for every single trip (i.e., OD-pair) does not yield the same result. A possible reason is that the OD-pair based calculation can only have an emission factor that varies according to the average speed, while the link-based emission calculation can vary the emission factor based on the average speed and street type.

#### 6.1.3. Link-Based and Correct Street Types vs. Link-Based and One Street Type

Table 5 shows that choosing “URB/Trunk-City/50/” as the only street type instead of the correct street type can underestimate the emissions by half, or overestimate them by 19% depending on the pollutant.

Which option should be chosen depends on a variety of factors including the accuracy and availability of the maximum speed data or street type data for the street network.

If a street with a speed limit of 50 km/h is assumed to be a street with a speed limit of 100 km/h, then the free-flow speed of 50 km/h would be considered a saturated flow for a street with a speed limit of 100 km/h. Hence, the link-based emissions should only be calculated if the correct street type is known for essentially all relevant streets of the network. Some of the maximum speeds in the OSM street network are noted in km/h while others are in mph, almost 3000 links have a speed limit of 200 km/h and another 1100 links have no speed limit in NYC. Hence, a link-based calculation might not be the best option when using an OSM street network in NYC.

## 7. Discussion

In the first macroscopic simulation (i.e., “On-Demand Meal Delivery (OD-Pairs)”), the mean of the average speeds ranges from 7 km/h (−40% demand) to 22 km/h (demand + 40%) when the traffic volume is varied. When the traffic volume is increased or decreased by 40%, the emission factors change by 10% to 30%. Given that the average speed in NYC varies throughout the day in the range of 9.5 km/h to 19.4 km/h, the emission factor should be increased or decreased by 5% to 10% depending on the pollutant. Of course, the appropriate increase and decrease of the emission factors should be determined for each study to ensure it is as accurate as possible. For the traffic congestion experienced in the simulation in this paper, the emission factor increases by up to 21% depending on the pollutant (i.e., CO_2_, one restaurant, all meals).

Direct deliveries from 128 restaurants produce three to four times higher emissions compared with a delivery tour every 15 min from 128 restaurants. Using the correct street type instead of “URB/Trunk-City/50/” either increases or reduces the emission factor depending on the pollutant. For example, depending on the traffic demand, the NO_x_ emissions per km increase by between 13% to 17% whereas the PM_10_ reduces by 5% to 7%.

## 8. Future Work

Future work should consider the use of electric vehicles for the delivery of meals. While these vehicles will not increase the emissions produced within the city, they increase the congestion; therefore, the emissions of other vehicles as well as increase the emissions elsewhere (i.e., energy production, production of batteries). Inconsiderately parked delivery vehicles (e.g., second row parking) might hinder the traffic flow, which has not been considered in this study.

## 9. Conclusions

Macroscopic simulations of New York City (NYC) in PTV VISUM to highlight the effect delivery trips have on the traffic flow as well as on the emissions produced within NYC have been reported in this paper. The study shows that the emission factor needs to be adjusted based on traffic volume. Hence, studies that ignore the impact of delivery vehicles on the traffic flow will calculate incorrect results. The same applies to comparing out-of-hour with daytime deliveries due to differences in the traffic volumes. The change in traffic flow will most likely affect the emissions of the delivery vehicles. In short, longer delivery trips increase the total emissions due to: (i) the increased trip lengths, (ii) the increased congestion increasing the emission factors and (iii) detours to avoid the congestion increasing the trip length. The latter two affect all vehicles’ emissions, not just the delivery vehicle’s emissions. Hence, the study highlights the importance of adjusting the emission factor to account for this. Decreasing the density of the restaurant network or increasing the mode share for cars increases the congestion, and therefore, the emission factors. The paper also highlights again that on-demand meal delivery by cars has an effect on congestion and emissions. Therefore, policymakers should encourage the use of more sustainable modes of transport (e.g., bicycles or SADRs) to deliver meals. Given that on-demand meal deliveries in cities require generally only short trips, it would be perfect for bicycles or SADRs. These would also make inconsiderably parked delivery vans (i.e., second row parking), which block the traffic and cause congestion, a thing of the past.

## Figures and Tables

**Table 1:** Studies simulating freight transport in PTV VISUM.

	Street Network	Traffic/Freight Data	Emissions
Perera et al. [19]	OpenStreetMap (minor local roads were excluded)	real traffic volumes (Average Annual Daily Traffic—AADT)	Emission costs
Perera et al. [20]	OpenStreetMap (minor local roads were excluded)	real traffic volumes (Average Annual Daily Traffic—AADT)	Emission costs
Gorin et al. [21]	Constructed by authors	four-step model (based on workplaces of each vehicle capacity per zone)	-
Gupta et al. [2]	Constructed by authors	four-step model (based on origin and destination surveys)	CO, HC, NO_x_, PM, etc. ^a^
Tang et al. [12]	Map in VISUM, National Transport Model (NTpM) of Ireland	National Traffic Model (NTM) of Ireland and NTpM (NRA), Traffic count records	CO, CH_4_, NO_x_, PM, CO_2_
Martino et al. [22] Fiorello et al. [23]	Based on network model	Based on economy model and energy model	-
Grebe et al. [24]	Graphic Integration Platform, OpenStreetMap	Revealed preference and stated preference surveys	-
Roider et al. [25]	Austrian transport model	Various freight transport statistics, road toll, vehicle count	-
Savadogo et al. [26]	-	SIMBAD, FRETURB	CO_2_, CO, PM, NO_x_, VOC ^a^
Jacyna et al. [27]	Constructed by authors	average daily traffic (ADT) and nominal hourly traffic volumes	CO, HC, NO_x_,
Gnap et al. [28]	-	transport model of the Žilina self-governing region	-
Binh [29]	-	data from the General Statistic Office of Vietnam, survey of 144 export-import and logistics companies	-

^a^ volatile organic compounds. - unknown/not considered.

**Table 2:** Example demand matrix.

Origin/Destination	A	B	C
A	3	1	0
B	0	3	1
C	1	0	4

**Table 3:** Change in the emissions per km (link based vs. OD-pair based calculation) (one street type for both).

Pollutant	Traffic Demand				
	60%	80%	100%	120%	140%
HC	0.88	0.87	0.85	0.86	0.85
CO	0.87	0.89	0.90	0.91	0.92
CO_2_ (total)	0.88	0.85	0.83	0.82	0.82
NO_x_	0.88	0.86	0.85	0.86	0.86
PM_10_	0.88	0.87	0.86	0.86	0.86
PM_10_ (non-exhaust)	0.88	0.88	0.90	0.91	0.92

**Table 4:** Change in the emissions per km (link based vs. OD-pair based calculation) (correct street type).

Pollutant	Traffic Demand				
	60%	80%	100%	120%	140%
HC	1.00	0.97	0.93	0.92	0.94
CO	1.48	1.44	1.40	1.40	1.41
CO_2_ (total)	0.86	0.82	0.77	0.76	0.79
NO_x_	0.78	0.76	0.74	0.73	0.74
PM_10_	0.95	0.93	0.91	0.91	0.92
PM_10_ (non-exhaust)	0.80	0.78	0.77	0.77	0.77

**Table 5:** Change in the emissions per km (i.e., link based using “URB/Trunk-City/50/” as a street type vs. link based with the correct street types).

Pollutant	Traffic Demand				
	60%	80%	100%	120%	140%
HC	0.88	0.90	0.91	0.92	0.93
CO	0.59	0.62	0.64	0.65	0.66
CO_2_ (total)	1.02	1.04	1.05	1.06	1.07
NO_x_	1.13	1.14	1.15	1.16	1.17
PM_10_	0.93	0.93	0.94	0.94	0.95
PM_10_ (non-exhaust)	1.10	1.14	1.16	1.18	1.19

## Data Availability

Map data copyrighted OpenStreetMap contributors and available from https://www.openstreetmap.org (accessed on 3 August 2021). Parcel delivery data provided under the Open Government Licence v3.0 maintained by Greater London Authority online available on: https://data.london.gov.uk/dataset/key-performance-indicators-of-demonstrator-freight-delivery-performance-with-electric-vans-in-central-london (accessed on 3 February 2020).
